# The Mottled Capsid of the *Salmonella* Giant Phage SPN3US, a Likely Maturation Intermediate with a Novel Internal Shell

**DOI:** 10.3390/v12090910

**Published:** 2020-08-19

**Authors:** J. Bernard Heymann, Bing Wang, William W. Newcomb, Weimin Wu, Dennis C. Winkler, Naiqian Cheng, Erin R. Reilly, Ru-Ching Hsia, Julie A. Thomas, Alasdair C. Steven

**Affiliations:** 1 Laboratory for Structural Biology Research, NIAMS, NIH, Bethesda, MD 20892, USA; Bing.Wang@nyulangone.org (B.W.); william.newcomb@nih.gov (W.W.N.); weimin.wu@nih.gov (W.W.); dennis.winkler@nih.gov (D.C.W.); Naiqian.Cheng@nih.gov (N.C.); stevena@mail.nih.gov (A.C.S.); 2 NYU Langone Health, CryoEM Core Facility, Division of Advanced Research Technologies, New York, NY 10016, USA; 3 NCI, NIH, Frederick, MD 21701, USA; 4 Advanced Imaging Core, NIDCD, NIH, Bethesda, MD 20892, USA; 5 Thomas H. Gosnell School of Life Sciences, Rochester Institute of Technology, Rochester, NY 14623, USA; err4592@rit.edu; 6 Electron Microscopy Core Imaging Facility, University of Maryland School of Dentistry, Baltimore, MD 21201, USA; RHsia@umaryland.edu

**Keywords:** cryoEM (cryo-electron microscopy), bacteriophage, virus, single particle analysis, 3D reconstruction, scaffold, ejection proteins

## Abstract

“Giant” phages have genomes of >200 kbp, confined in correspondingly large capsids whose assembly and maturation are still poorly understood. Nevertheless, the first assembly product is likely to be, as in other tailed phages, a procapsid that subsequently matures and packages the DNA. The associated transformations include the cleavage of many proteins by the phage-encoded protease, as well as the thinning and angularization of the capsid. We exploited an amber mutation in the viral protease gene of the *Salmonella* giant phage SPN3US, which leads to the accumulation of a population of capsids with distinctive properties. Cryo-electron micrographs reveal patterns of internal density different from those of the DNA-filled heads of virions, leading us to call them “mottled capsids”. Reconstructions show an outer shell with T = 27 symmetry, an embellishment of the HK97 prototype composed of the major capsid protein, gp75, which is similar to some other giant viruses. The mottled capsid has a T = 1 inner icosahedral shell that is a complex network of loosely connected densities composed mainly of the ejection proteins gp53 and gp54. Segmentation of this inner shell indicated that a number of densities (~12 per asymmetric unit) adopt a “twisted hook” conformation. Large patches of a proteinaceous tetragonal lattice with a 67 Å repeat were also present in the cell lysate. The unexpected nature of these novel inner shell and lattice structures poses questions as to their functions in virion assembly.

## 1. Introduction

“Giant” phages—also known as “jumbo” phages—have genomes in excess of 200 kbp dsDNA [1], and correspondingly complex assembly pathways. One such phage, the *Salmonella* phage SPN3US, packages a 240 kbp dsDNA genome into a capsid ~1460 Å in diameter (vertex-to-vertex), followed by an attachment of a contractile tail [2,3]. The head is composed of ~50 proteins, with three being highly abundant: the major capsid protein, gp75, and the two ejection proteins, gp53 and gp54 [3]. Collective phage lore suggests that capsid assembly should commence with the formation of a procapsid guided by interactions between the growing surface shell and a morphogenic scaffold or core [4,5]. This is followed by activation of the protease and cleavage of several proteins, leading to the expulsion of scaffolding protein(s) and the major structural transformation (usually expansion) of the surface shell.

In the current paradigm for capsid maturation, the main morphological differences between a procapsid and its mature version are that the procapsid shell is thicker-walled, rounder and smaller—typically by 10% to 20%. There are, however, exceptions to this trend. For instance, the procapsid of herpes simplex virus (HSV)—an animal virus with many phage-like properties, indicative of common ancestry [6]—changes little in size as it matures, although it does change markedly in sphericity [7]. Our a priori expectation for giant phages such as SPN3US is that they share the basic maturation features of smaller phages, some additional features of larger phages such as T4 (which assembles its procapsid on the host cell membrane [8,9]), and some additional—probably more complex—mechanisms exclusive to giant phages.

We started by imaging virion heads by cryo-EM, obtaining a reconstruction with a T = 27 icosahedral outer shell very similar to other giant phages, such as ΦKZ [10], PBS1 [11] and RSL1 [12]. To probe the SPN3US head assembly pathway, we analyzed the composition and structure of precursor capsids isolated from infections with a protease-deficient mutant by gel electrophoresis and cryoEM (cryo-electron microscopy). These capsids are visibly filled with a material less dense than DNA, presumably protein, that we describe as “mottled”. Three-dimensional reconstructions reveal an icosahedral two-shell particle, with a T = 27 outer shell similar to the virion head, and an inner T = 1 shell composed of the ejection proteins gp53 and gp54. This inner shell is not evident in the mature virion reconstruction, but the ejection proteins are still present, indicating that the inner shell is drastically altered by proteolysis and DNA packaging.

## 2. Materials and Methods

### 2.1. Purification of Wild-Type Virions and Mutant Capsids.

Wild-type virions were prepared as described in [3]. The SPN3USprotease mutant *245*(am59) was propagated in the non-permissive strain of *Salmonella enterica* Typhimurium T9079 in LB+N broth (lysogeny broth with 0.2% nutrient broth) supplemented with 2 mM CaCl_2_ and 2 mM MgCl_2_. An overnight culture of Salmonella was sub-cultured (1:75) into 400 mL of LB+N, shaken at 35 °C until it reached an OD600 of ~0.4 and infected with *245*(am59) (MOI of 10). At 25 min post-infection, cells were harvested (5000 g, 5 min, 21 °C) and resuspended in fresh LB+N to remove any remaining input phage. After an additional 2 h of shaking at 35 °C, the cells were harvested (3000 g, 5 min, 21 °C). The cell pellet was resuspended in 2 mL of HEPES buffer (200 mM HEPES—Alfa Aesar, 100 mM NaCl, 10 mM MgSO_4_, pH 7.5, Ward Hill, Massachusetts, USA) supplemented with lysozyme (8 mg, Thermo Fisher, Waltham, MA, USA) and DNAase (100 U, Roche, Basel, Switzerland) and incubated for 30 min at room temperature with gentle rocking. The lysate was clarified by centrifugation (5250 *g*, 10 min, 21 °C), giving a supernatant called the “8k supe”. The 8k supe was fractionated using a 20–60% sucrose density gradient centrifuged at 22k for 1 h at 4 °C to yield three bands: band 1 with phage tails and other proteins, band 2 with empty capsids, and band 3 with mottled capsids.

### 2.2. Electron Microscopy

Samples of virions were prepared on Quantifoil grids and imaged on film in a CM200 electron microscope operated at 120 kV. Samples of the 8k supe and sucrose gradient fractions were prepared on thin continuous carbon overlaying a lacy carbon support. Micrographs were recorded on a JEOL electron microscope operated at 200 kV or a Krios electron microscope at 300 kV, both with energy filters (Table S1). Additional details are provided in the Supplementary Material.

### 2.3. Image Processing

Five data sets were collected (Table S1). Data set 1 of whole virions was processed with EMAN2 [13], yielding a head reconstruction at ~30 Å. The processing of data sets 2–5 was performed using Bsoft [14] and the Peach system to distribute jobs across a cluster [15]. Map interpretation included calculating symmetry-adjusted radial profiles, estimating capsid content distributions from single particle reconstructions and segmenting the inner and outer shells of the mottled capsid. The tetragonal lattice sheets found in the 8k supe were processed using the 2D crystallography tools in Bsoft [14]. The following maps were deposited at the Electron Microscopy Databank: Fixed mottled capsid—EMD-22332; Empty capsid—EMD-22331; Mottled capsid—EMD-22333. Additional details are provided in the Supplementary Material.

## 3. Results

### 3.1. The Mature Virion Head

**Figure 1:**
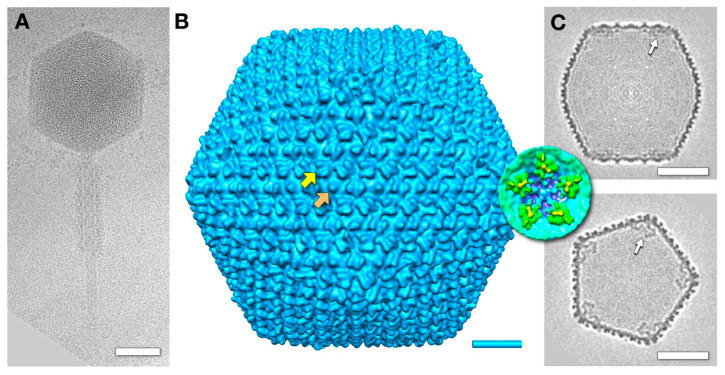
The mature virion of *Salmonella* phage SPN3US. (**A**) A virion with a DNA-filled head and a tail with a contracted sheath. Scale bar: 500 Å. (**B**) A reconstruction of the virion head showing the T = 27 architecture. Each pair of capsomers is connected by “wings” (yellow arrow), and each hexamer has a central protrusion (orange arrow). Scale bar: 200 Å. (**C**) A central slice along a 2-fold view (top) and a slice along a 5-fold view (bottom) through the reconstruction. Organized densities that we call “antlers” are evident under the 5-fold vertices. Inset: An isosurface rendering of the antlers from the inside of the capsid. Scale bar: 500 Å.

CryoEM of the SPN3US virion revealed a head of ~1460 Å (vertex-to-vertex), with a striated or punctate pattern (depending on orientation) of the packaged DNA, which has a distinctive 25 Å spacing (Figure 1A). Previous proteomic analyses of a tailless mutant determined that, in addition to its DNA, the SPN3US head also contains a substantial amount of proteins with still obscure functions [2,3]. Many of the SPN3US head proteins have homologs to head proteins in the related *Pseudomonas aeruginosa* phage ΦKZ [2]. In ΦKZ, a copious amount of internal proteins form an unusual “inner body” structure encapsidated with the DNA [16,17]. To ascertain whether a similar structure exists within the SPN3US head, we performed bubblegram imaging on virions. In this technique, specimens are subjected to higher levels of electron irradiation than are normally used in cryo-EM, eliciting the formation of bubbles of hydrogen gas at the sites of DNA-embedded proteins [17,18,19].

**Figure 2:**
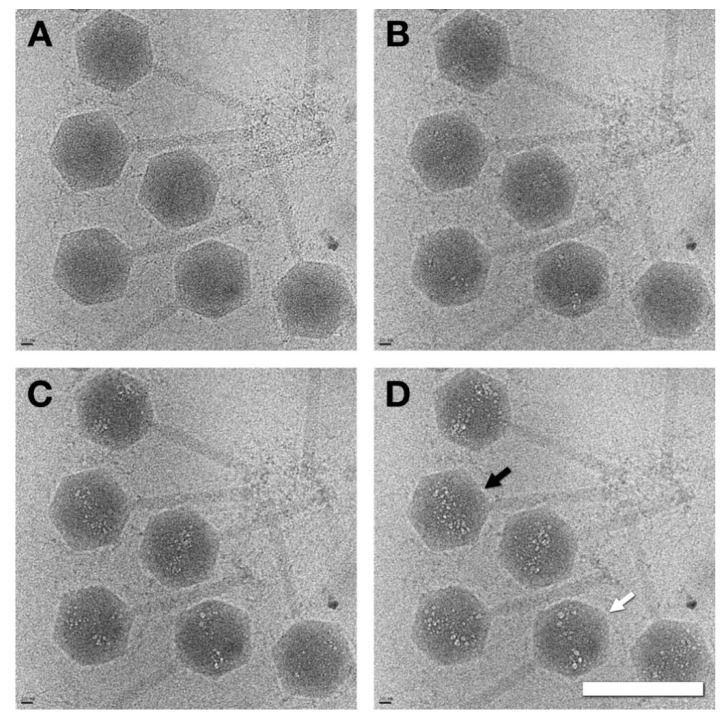
Dose series of SPN3US bubblegram images. (**A**) Low dose image (1st exposure). (**B**–**D**) Exposures 8–10. The bubbles come from irradiated proteins inside the capsid. One in this image, marked with a black arrow, appears cylinder-like, possibly due to its orientation. Others show dipolar distributions, e.g., white arrow. Scale bar: 2000 Å.

Dose series micrographs of SPN3US virions showed definite bubbling patterns within the heads, implying that proteins are present in substantial amounts. The bubbling patterns varied somewhat from particle to particle, but a common motif was a bipolar distribution (Figure 2). We conclude that although SPN3US virions contain substantial amounts of internal proteins, they are not organized in the same way as the ΦKZ inner body [17]. While many homologous head proteins are shared between ΦKZ and SPN3US, there is considerable variability in the head protein content of the two phages, especially with regard to the numbers and amounts of proteins belonging to two different paralog families [3].

Our reconstruction of the SPN3US nucleocapsid, calculated to ~30 Å resolution, shows it has a T = 27 icosahedral architecture (Figure 1B) similar to those of other giant phages such as ΦKZ [10] and PBS1 [11]. The heads of all these giant phages are much larger than that of the prototype HK97, with its T = 7 capsid [20,21]. The cores of each of the capsomers in the SPN3US capsid are composed of a typical HK97 fold oligomer, with external “wings” forming pairwise connections between capsomers (yellow arrow in Figure 1B). Each hexamer has a small, central protrusion (orange arrow in Figure 1B), whereas there is a central hole in each of the pentamers that form the icosahedral vertices. The central structure on the SPN3US’s hexamer is reminiscent of the *hoc* protein that decorates the hexamers of the T4 phage shell [22].

Slices through the reconstruction of the SPN3US capsid showed there were densities under the 5-fold vertices (white arrows in Figure 1C and inset) that project inwards towards the 2-fold axes. These “antlers” are more prominent than similar densities reported under the vertices of other giant phages [11]. The identity of the protein(s) forming these structures remains to be determined.

### 3.2. Capsids Produced with Impaired Protease Activity

**Figure 3:**
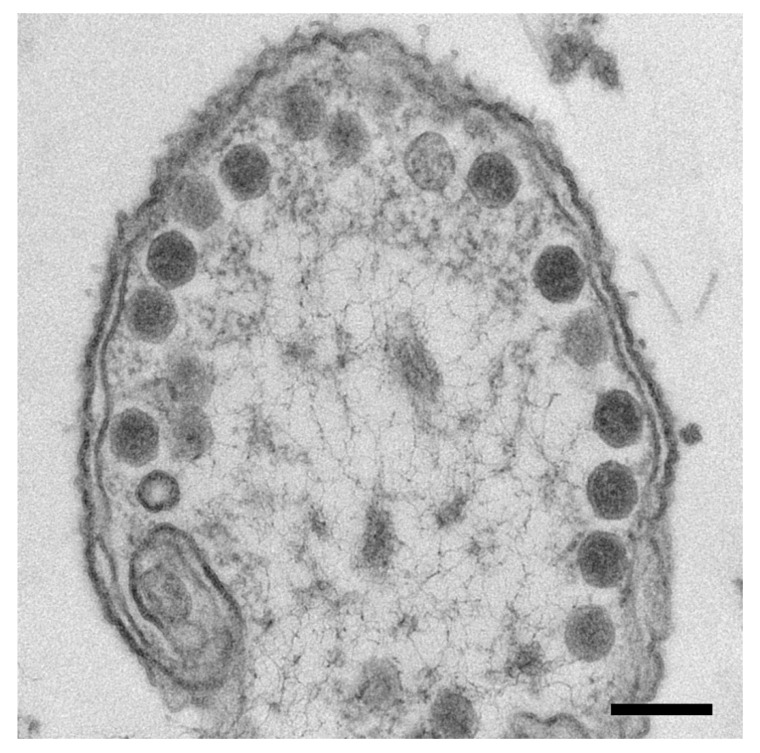
Micrograph of a plastic thin section of a *Salmonella* cell with SPN3US capsids lined up at the cell membrane. Scale bar: 2000 Å.

Based on precedents in HSV1 [23] and dsDNA phages [24,25], we expected that curtailing the viral protease activity would lead to an accumulation of maturational precursors. To this end, we employed an amber mutant (*245*(am59)) that codes for a defective protease under non-permissive conditions. Electron microscopy of plastic thin sections of *245*(am59)-infected cells indeed showed abundant particles lining the cell membrane, which we interpret as different types of capsids (Figure 3). Many of them project polyhedral profiles that we associate with a mature-like capsid. The staining extends across the interior of most of the capsids, indicating that they contain some material, which we take to be protein as DNA is not packaged in the absence of viral protease activity.

Since, generally, phage precursor capsids are structures susceptible to damage, we tried to minimize the amount of handling of the *245*(am59) samples. Our starting point was always a lysate of am59-infected cells clarified by low-speed centrifugation (referred to as the “8k supe”). In one set of experiments, the 8k supe was lightly fixed with glutaraldehyde, while in other experiments fixation was not employed (see Supplementary Material for detailed protocols).

**Figure 4:**
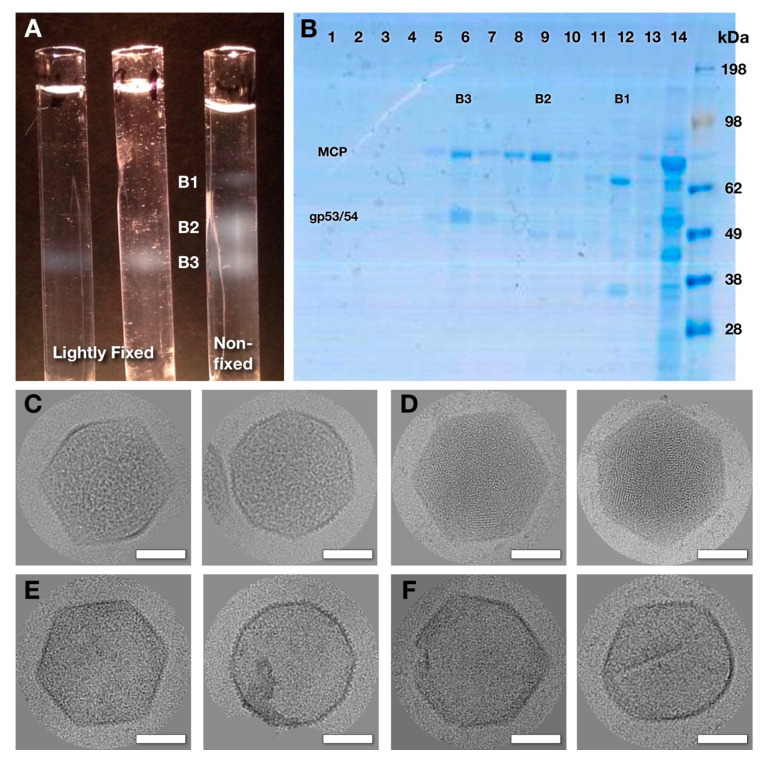
Purification and imaging of SPN3US capsids from a protease-impaired mutant. (**A**) Tubes after sucrose density centrifugation of the 8k supe showing 1 band (left two tubes) of light-scattering material for the sample lightly fixed with glutaraldehyde (1.5 and 3 mM respectively), or 3 bands (right tube) for the unfixed sample. (**B**) SDS-PAGE gel of fractions (1: bottom/14: top) from the unfixed gradient in (A). The uncleaved major capsid protein (MCP) of the mottled (band B3) and empty (band B2) capsids is prominent, as is an additional doublet of bands of the uncleaved ejection proteins (gp53/54) for the mottled capsid. Lane 12 (band B1) has a strong band for the tail sheath protein and lane 14 contains numerous proteins at the top of the gradient. Examples of capsids from cryo-electron micrographs: (**C**) mottled (band B3), (**D**) DNA-filled (band B3), (**E**) empty (band B2) and (**F**) broken and distorted empty (band B2). Scale bars: 500 Å.

When we first observed the 8k supe via electron microscopy, we found capsids and many other proteins and structures (including easily identifiable phage tails and bacterial flagella). Our primary aim was to further purify the capsids so as to determine their composition and structure. The 8k supe was subjected to sucrose density gradient centrifugation and fractionated. In Figure 4A, the 8k supe used in the two tubes on the left was lightly fixed with glutaraldehyde before centrifugation, while the tube on the right was spun without fixative. With the fixative, the material in the 8k supe yielded a single density band (B3) and was clearly cross-linked, while without the fixative two slower-migrating bands appeared (B1, B2). Gel electrophoresis indicated that the uppermost band (B1) contained mostly phage tails and other proteins, while the lower two bands contained the major capsid protein, gp75 (Figure 4B). In the slower sedimenting band (B2), the only major protein is gp75, the major capsid protein, while in the lowest band (B3), the three major proteins are gp75 and the two ejection proteins gp53 and gp54.

CryoEM of both the fixed and unfixed band B3 samples clearly showed capsids with internal content (Figure 4C). We also found a small number (~0.5%) of DNA-filled capsids (Figure 4D) that likely originated from the virus used for infection. The internal material of the capsids in Figure 4C has a different pattern from the typical striated and punctate patterns of DNA-filled capsids. Because of their distinctive appearance, we refer to the abundant B3 capsids as “mottled”. CryoEM of the band B2 sample showed empty or close-to-empty capsids (Figure 4E), or, to a lesser extent, broken or deformed particles (Figure 4F). We conclude that these are mottled capsids that lost their content to a greater or lesser extent during purification, and do not originate from a form of the capsid occurring inside the cell. The crosslinking therefore captures the mottled capsid in a more representative in vivo state.

### 3.3. Three-Dimensional Reconstructions

**Figure 5:**
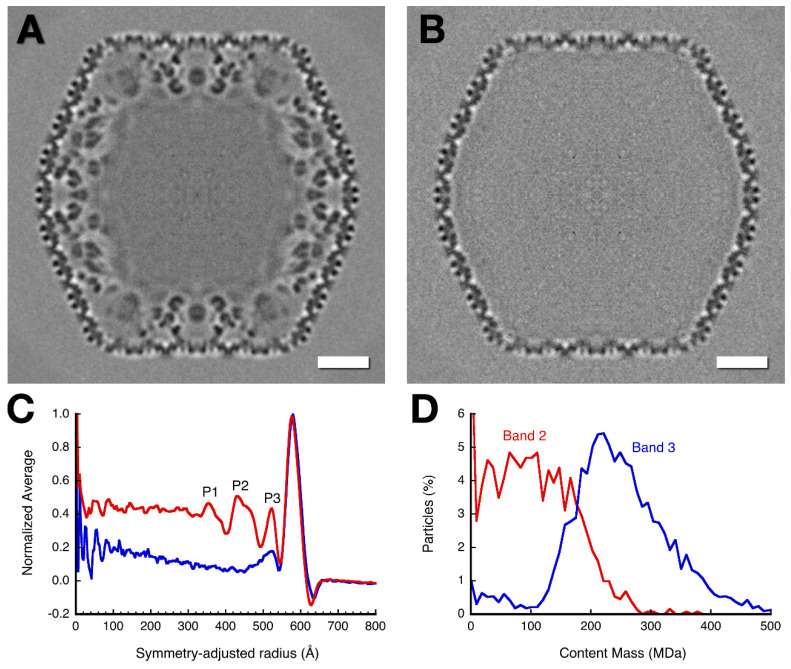
Central sections of maps of mottled (**A**) and empty (**B**) capsids, reconstructed from particles purified from bands 3 and 2 in Figure 2A, respectively. (**C**) Symmetry-adjusted normalized radial profiles of the mottled (red) and empty (blue) maps. Labels 1–3 mark peaks in the inner shell density. (**D**) Content distribution histograms for the particles from band 2 (*n* = 1322) and band 3 (*n* = 3319) determined from an icosahedral reconstruction of each particle. The content mass was calibrated assuming an outer capsid mass of 136 MDa.

In a preliminary reconstruction using particles (unfixed) from the 8k supe, an ab initio reference map was generated using only icosahedral symmetry to initiate processing, yielding a final structure at ~30 Å resolution (Table S1, data set 2). In this map, a double-shell architecture was immediately evident, with a region inside the inner shell that appears to be filled with some amorphous density (Figure 5A). A subsequent reconstruction from a fixed sample yielded a map of the mottled capsid at 20 Å resolution (Figure S1A). Since the gradient separates two capsid species in bands 2 and 3 with different compositions in the unfixed samples (Figure 4B), we also reconstructed capsids from those bands. This yielded reconstructions of the mottled capsid from band 3 at 16 Å resolution (Figure 5A and Figure S1) and the empty capsid from band 2 at 19 Å resolution (Figure 5B, Figure S1). The outer shells of these capsids are very similar to that of the mature virion (see below). The biggest difference between the mottled and empty capsids is the presence of an inner shell in the former—a structure not seen before. It forms an open network with an intricate pattern when rendered as spherical shells (Figure S2). The gel analysis in Figure 4B indicated that the major constituents of the inner shell are the ejection proteins gp53 and gp54. Local resolution analysis of the mottled capsid (Figure S3) confirmed that the structure is well defined for the outer capsid shell, but less so towards the center. The open network structure of the inner shell may be intrinsically flexible, and with an active protease cleavage may further weaken the connections.

### 3.4. Mass Distribution in the Capsid Maps

The internal contents of the mottled capsid comprise the inner shell and a diffuse density in the central region. To estimate the total internal mass of the mottled capsid, we calculated the radial density distributions of unfixed mottled and empty capsid maps, adjusted for their angular shapes (Figure 5C). The radial distance indicated corresponds to the outer shell at the two-fold axis. The relative size of the peak associated with the outer shell and the level of background outside the capsid were used to normalize the profiles. The profile of the mottled capsid shows three peaks for the inner shell, and an almost constant density level for central amorphous matter. Only the outer shell peak is distinct in the profile for the empty capsid, with a small peak just inside it and much less internal content compared to the mottled capsid. Table 1 provides an analysis of the density distributions relative to the outer shell mass of 136 MDa. The nature of the amorphous density is not yet clear. It probably represents randomly distributed protein(s) without any order.

We also observed from perusal of the micrographs that the capsids vary in terms of their amount of content. To quantify the content on an individual basis, we calculated a reconstruction from each particle and integrated the internal density relative to outer shell and background. We then constructed a histogram that plots the number of particles with a specific internal mass. The result is shown in Figure 5D for the unfixed mottled and empty capsids. The peak for the mottled capsids is ~220 MDa, similar to the content estimated from the radial profile (Table 1). For the empty capsids, the peak is broad and centered at ~100 MDa, higher than the internal mass from the radial profile.

The internal volume of the mature capsid is ~8.6 × 10^8^ Å^3^, compared to the outer shell of ~2.5 × 10^8^ Å^3^. Given a typical protein density of 1.35 g/cm^3^ = 0.81 Da/Å^3^, the volume occupied by 220 MDa is ~2.7 × 10^8^ Å^3^, ~32 %. We analyzed 27 bona fide DNA-packaged heads from band 3 (Figure 2D) in the same way, obtaining an average density relative to the outer capsid shell of 1.09 ± 0.17 (standard deviation). This agrees with the visual inspection of packaged heads (Figure 1A and Figure 3D) wherein the outer shell has approximately the same density as the interior. The packaged heads therefore have an average content of 510 ± 80 MDa. This is consistent with the maximum in Figure 5D (red curve), which indicates that we included a few DNA-containing capsids in the particles extracted from data set 5.

Given an average mass per base pair of 660 Da, one copy of the genome of 240 kbp dsDNA has a mass of 158 MDa. Together with the 220 MDa of uncleaved proteins in the mottled capsid, that gives a total of 378 MDa, which is still much less than 510 MDa. Mass spectrometry of the virion head indicated numerous proteins in relatively high copy numbers [3], which could account for the additional mass compared to the mottled capsid.

### 3.5. The Outer Shell

**Figure 6:**
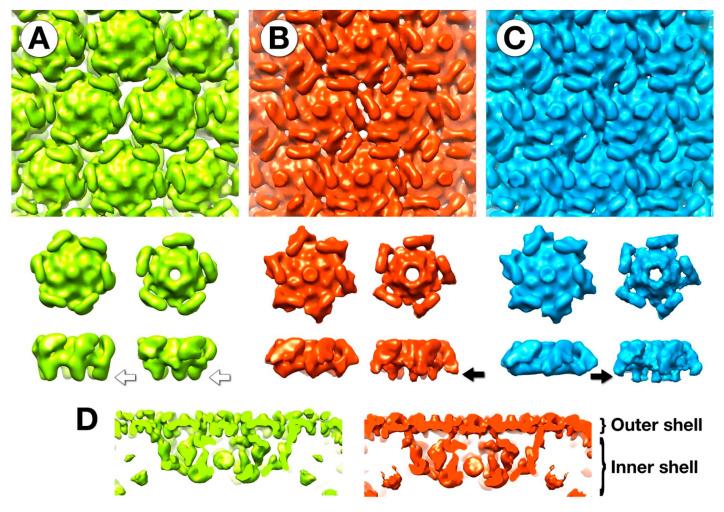
The outer shell of the (**A**) fixed mottled, (**B**) unfixed mottled and (**C**) empty capsids. The top row shows the progressive closure of the gaps (white spaces) between the capsomers. The rows below that show the top and side views of segmented hexamers and pentamers. The hexamer maps are the averages of 5 unique capsomers in the asymmetric unit. The fixed capsomers show inward protrusions (white arrows) that are absent in the non-fixed hexamers and displaced in the non-fixed pentamers (black arrows). (**D**) The capsomers in the fixed mottled map (**left**) have more connections to the underlying inner shell than the unfixed map (**right**).

The outer shell of the mottled capsid has the same T = 27 icosahedral symmetry as the mature virion (Figure 1B). As for the mature virion, the capsomers are connected by the externally protruding “wings”, and each hexamer has a central protrusion (compare Figure 1B and Figure 6). While the hexamers and pentamers are composed of the same protein, gp75, they differ in conformation (Figure 6). The core of the hexamer is flatter and the pentamer is more cone-shaped.

In the fixed mottled capsid, there are sizable gaps between the capsomers below the wings (Figure 6A). These appear much less prominent in the unfixed mottled capsid (Figure 6B) and are mostly closed off in the empty capsid (Figure 6C). This is reminiscent of the closure of the herpesvirus capsid floor upon maturation [7].

On closer examination, the inward extensions or “feet” of the hexamers of the fixed mottled capsid (white arrows in Figure 6) protrude more than those in the other two maps. The feet are plausible connections to the inner shell. Without fixing these connections in place, they can sever, allowing the inner shell to dissociate, and in some cases extrude, yielding empty capsids. The maturation of the capsid in the absence of proteolytic cleavage has precedents in the cases of HSV1 [7] and the T4 polyheads (tubular capsid analogs, [26]). A comparison of the unfixed empty and mottled capsids with the mature capsid in the virion shows little difference. This suggests that the N-terminal part of the major capsid protein removed by the maturational protease [3] is not visible in these density maps, and may be disordered.

### 3.6. Segmentation and Analysis of the Inner Shell

**Figure 7:**
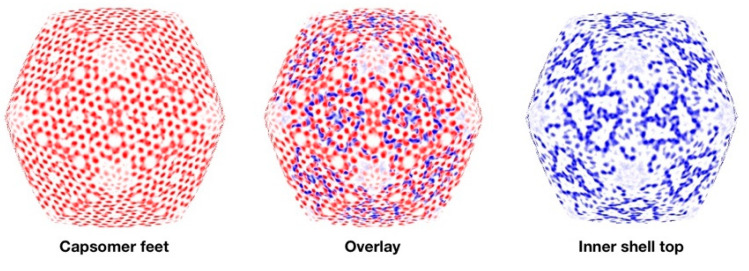
Connections between the inner and outer shells. The left shows a spherical section at the feet of the outer shell capsomers (radius ~590–600 Å), the right shows the inner shell ~20 Å deeper, and the middle is an overlay superimposing the likely connecting parts.

Spherical sections through the fixed mottled capsid reveal complex transitions between the layers in the particle (Figure S2). Figure 7 (left) shows a spherical section of the “feet” layer, indicating that it is the major structural feature projecting towards the inner shell. About 20 Å inward from this section, the inner shell has a markedly different appearance (Figure 7, right). The overlay of the feet and uppermost inner shell densities (Figure 7, middle) emphasizes the symmetry mismatch between the two shells.

**Figure 8:**
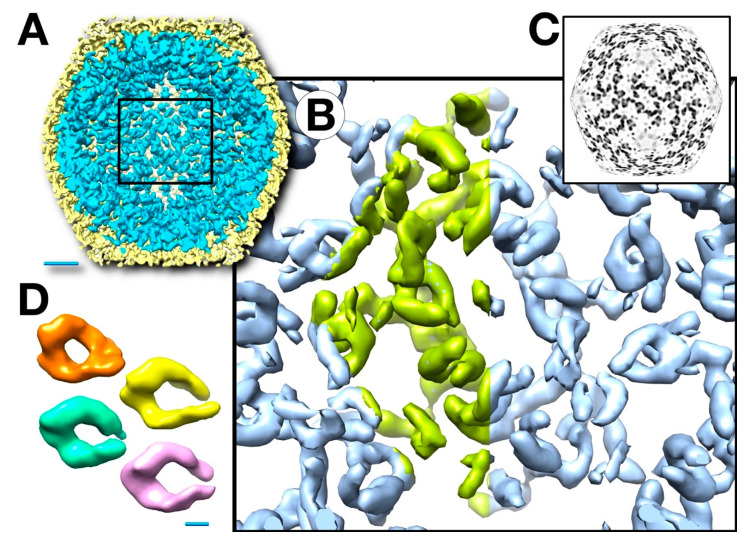
The T = 1 inner shell of the SPN3US capsids from the protease mutant *245*(am59) viewed from inside the capsid (**A**), enlarged with the asymmetric unit in green (**B**) and a spherical section (**C**). (**D**) Several densities show a twisted hook shape (left) while others are elongated. Scale bars: A: 200 Å; D: 20 Å.

The inner shell is an open network of proteins connected well enough to follow T = 1 icosahedral symmetry. Based on our SDS-PAGE results (Figure 4B), these densities are mostly composed of the ejection proteins gp53 and gp54. Some of these densities have a “twisted hook” shape (Figure 8D), while others are elongated. We count 11 to 12 of the former densities in the asymmetric unit (Figure S4). The exact number is difficult to specify because the resolution falls off towards the middle of the capsid (Figure S3). The conformations of the individual hooks also vary, suggesting substantial flexibility. We conclude that the densities in the inner shell represent the different conformers of the ejection proteins. Estimating the mass of a hook is complicated by the varying resolution and the arbitrary choice of a threshold to determine the volume. However, a hook is too large for one 45 kDa ejection protein, and may contain two to four copies. This means there are 1440–2880 copies of gp53 and gp54 combined per mottled capsid, with the lower number close to the >1200 previously estimated (conservatively) by the mass spectrometry of the virion head [3]. The corresponding mass is 65–130 MDa, a fraction of the mass estimated in Table 1. The mass spectrometry analysis also indicated that many other proteins are present, albeit in smaller amounts, which could account for the remaining mass. From SDS-PAGE (Figure 4B), there was little or no evidence of gp22, a protein that is abundant in infected cells [27].

### 3.7. Analysis of a Tetragonal Sheet Observed in the 8k Supe

**Figure 9:**
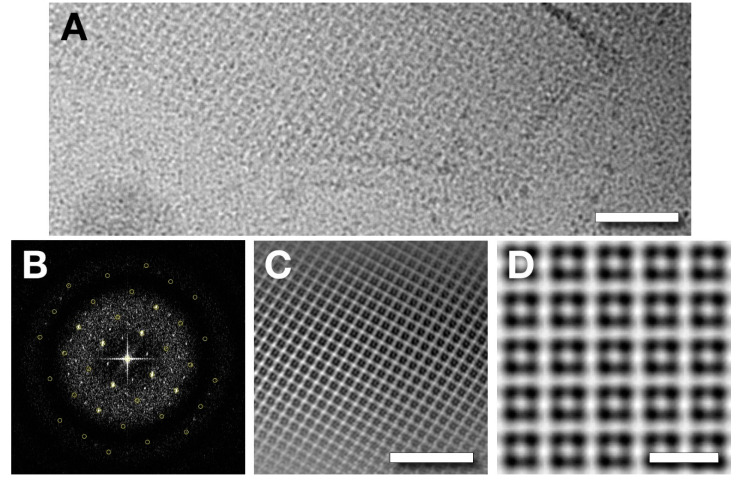
(**A**) A sheet of protein found in the 8k supe (clarified cell lysate). Scale bar: 500 Å. (**B**) Diffraction pattern from a sheet, showing a square unit cell with a 66.8 Å lattice spacing and reflections out to ~20 Å. (**C**) A Fourier filtered image of a sheet. Scale bar: 500 Å. (**D**) A reconstruction of the lattice from one class average derived from unit cells extracted as single particles. Scale bar: 100 Å.

Our micrographs of the cell lysate (8k supe, data set 2) revealed macromolecular complexes of several kinds other than capsids. In addition to flagella and unattached tails (not shown), these samples had large patches of periodic lattice (Figure 9A). Further processing indicated a tetragonal (p4) unit cell with a lattice spacing of ~67 Å (Figure 9B), giving the lattice in Figure 9C after Fourier filtering. To obtain more detailed information, we attempted a single particle approach, whereby each unit cell was extracted as a particle and aligned. The result was propagated into a lattice with the appropriate spacing (Figure 9D). These assemblies cannot be derived from the capsids of infecting phages because their symmetry and lattice constant are not consistent with what would be expected for a ruptured capsid derived from an infecting virion (p6 symmetry and 145 Å repeat). In addition, these sheets were found in a lysate from a mutant infection. The nature of these sheets and their role in viral propagation still need to be investigated. Of note, a proteinaceous sheet has been proposed to enclose a cytoplasmic compartment in *Pseudomonas chlororaphis* infected with the giant phage 201Φ2–1 [28].

## 4. Discussion

We set out to find intermediates in the capsid assembly of the *Salmonella* phage SPN3US, exploiting an amber mutant in its protease gene. In so doing, we isolated the mottled capsid, which has an outer shell very similar to that of the mature virion and other giant phages [10,11]. It also has an elaborate inner shell that has a novel structure not—to our knowledge—seen before. This structure may serve as a scaffold for assembly. Our primary interest is to understand what stage of assembly and maturation this capsid represents, and what it means for the final virion structure.

### 4.1. The Outer Capsid Shell

The procapsids of herpesviruses [7] and HK97-related phages [20,29,30,31] have spherical or near-spherical shapes. We therefore expected the SPN3US procapsid to be roughly spherical, and observed many particles with a round appearance in plastic sections (data not shown). However, the mottled capsid reconstruction has a decidedly angular shape (Figure 5A), similar to the mature capsid (Figure 1B,C). While a projection down the 5-fold axis of the map has a spherical appearance, it does not account for the prevalence of round particles seen in plastic sections. The mottled capsid is therefore unlikely to be the first assembly product or procapsid.

Our use of a fixative to purify capsids from a cell lysate was intended to mitigate the effects of forces that may affect the structure. Indeed, without a fixative, the mottled capsids tend to lose their contents (Figure 4), and the empty capsids appear to be more fragile. We conclude that empty capsids are rare in cells, and are an artifact of purification.

The outer shell itself appears to undergo a moderate conformational change, from the open structure of the fixed mottled capsid to a more closed structure in the unfixed mottled and empty capsids (Figure 6). The main aspect of the closure in the mottled capsid is the retraction of the capsomer feet, severing the connections with the inner shell and allowing it to be released. These connections are the likely locations of the N-terminal 65 residues of the major capsid protein that is removed by the maturational protease [3].

### 4.2. Major Internal Proteins

Functionally, the internal proteins of giant phages are expected to be of two kinds: scaffolding (or core) proteins and ejection (or cargo) proteins. In the mottled capsid, we found the ejection proteins gp53 and gp54 (Figure 4B). The densities under the vertices in the mature head (Figure 1C) could be remnants of the inner shell. However, they do not overlap with any densities of the inner shell in the mottled capsid. In fact, we did not find evidence that any part of the inner shell structure remains as such in the mature capsid, although its main constituents gp53 and gp54 are retained. It follows that remodeling on a major scale takes place, concomitant with DNA packaging. Our analysis further shows that there is more than enough space within the capsid for both the genome and all the inner shell proteins.

### 4.3. Minor Proteins

While we assign the ejection proteins as the predominant internal components, there are also indications of the likely disposition of other capsid-associated proteins. These include: (i) An additional protein bound on the outer surface at the center of each hexamer in the outer shell, making 260 copies per capsid (Figure 1B and Figure 6). This mode of binding is reminiscent of the hoc protein of T4 [22]. (ii) The mature virion has approximately 12 copies of the pentameric “antler” complexes underlying the vertices, whereat each protomer may encompass several different subunits (Figure 1C). (iii) A substantial amount of protein is incorporated in a disordered fashion into the central “amorphous zone” (Table 1). The presence of this protein is attested by the bubblegrams (Figure 2) and the “above background” level of density present in this region in the reconstructions (Figure 5A,C). While most, if not all, of the encapsidated proteins appear to be destined for delivery to a host cell, their natures, activities and trajectories in assembly remain to be determined.

### 4.4. The Mottled Capsid—A Maturation Intermediate?

How close is the mottled capsid to the initial assembly product, the procapsid? The mottled capsid is highly angular, lacking the spherical shape of the procapsids of other viruses with the HK97 fold. If the mottled capsid is not the procapsid, it nevertheless has many features that at least place it early in the maturational pathway.

A common theme is that the capsomers of the procapsid are loosely bound, maturing into a more stable, interlocked final structure [7,32,33]. The gaps between the capsomers spontaneously close when not constrained in the unfixed capsids (and the mature virion). In our case this happened during purification, while in vivo it is accelerated by the maturational protease. The capsid transformations can occur in the absence of [7] or before DNA packaging [34]. The mottled capsid may therefore be an intermediate that has already undergone some maturation steps.

Many procapsids undergo changes in size (expansion in the case of HK97-like systems) and wall thickness as they mature. However, the mottled capsid of SPN3US is the same size as the mature capsid, similar to the herpesviruses, where there is also negligible change in size [7]. The outer shell is somewhat thicker in the fixed mottled capsid (Figure 6) than in the unfixed and mature capsids, but not to the extent seen in other phages [31,35].

The three major proteins in the mottled capsid, gp75, gp53 and gp54, follow a similar production profile [27], consistent with their co-incorporation. The assembly could therefore be similar to that of the herpesviruses, whereby the major capsid and scaffold proteins are incorporated together [36,37]. How the mottled capsid assembles is complicated by the symmetry-mismatch between the outer and inner shells (Figure 7).

### 4.5. Future Research

In these studies, we determined capsid structures for a giant phage in both its mature state and as the product of a mutant infection in which proteolytic maturation and DNA packaging were blocked. The latter particles comprise two nested shells: the outer one has an HK97-like architecture with T = 27 symmetry, while the inner one is an open network of proteins with T = 1 symmetry. These observations raise fundamental questions to be addressed in future research, including the following: What is the first-assembled procapsid? How many metastable transition states are encountered on this pathway? More specifically, how does the SPN3US T = 1 shell form and then transform during proteolysis and DNA packaging, as we infer it must, as its major components, gp53 and gp54, relocate into the interior of the mature head? Finally, which protein forms the regular tetrameric lattice observed in cell lysates? Is it present in wild type infections, and if so, what is its role?

## Figures and Tables

**Table 1:** Estimated densities of the empty and mottled capsids based on their symmetry-adjusted normalized radial profiles.

Peak Label	Empty Capsid		Mottled Capsid	
	Peak Position	Mass (MDa)	Peak Position	Mass (MDa)
Amorphous mass	<450	30	<334	60
P1			355	40
P2			432	81
P3	523	29	523	47
Outer shell	579	136 ^†^	576	136 ^†^

† The main peak is the outer shell composed of 1615 copies of the 84 kDa major capsid protein (in its uncleaved precursor state and assuming one vertex for the portal), giving a total mass of 136 MDa.

## Supplementary Materials

The following are available online at https://www.mdpi.com/1999-4915/12/9/910/s1, Table S1: Image processing report for the DNA-filled mature capsid, and the various forms of the mottled capsid; Figure S1: Reconstruction details: Fourier shell correlation and particle orientation distributions; Figure S2: Icosahedral-adapted spherical sections of the fixed mottled capsid reconstruction; Figure S3: Isosurfaces of the unfixed mottled capsid reconstruction colored by local resolution estimates.; Figure S4: Segmentation of the inner shell of the fixed mottled capsid reconstruction, with symmetry-related densities colored the same.
