# Supplementary material for: The Mottled Capsid of the Salmonella Giant Phage SPN3US, a Likely Maturation Intermediate with a Novel Internal Shell
Source: Viruses. 2020 Aug 19;12(9):910. doi: 10.3390/v12090910 (PMC7552025; doi:10.3390/v12090910)
Supplement: Supplementary file 1 [file viruses-12-00910-s001.zip › viruses-889479-supplementary.pdf]

August 19, 2020

Supplementary material

The mottled capsid of the *Salmonella* giant phage SPN3US, a likely maturation intermediate with a novel internal shell

J. Bernard Heymann[1], Bing Wang[1,3], William W. Newcomb[1], Weimin Wu[1,5], Dennis C. Winkler[1,4], Naiqian Cheng[1†], Erin R. Reilly[2], Ru-Ching Hsia[6], Julie A. Thomas[2], Alasdair C. Steven [1]

[1] Laboratory for Structural Biology Research, NIAMS, NIH, Bethesda, MD 20892.
[2] Thomas H. Gosnell School of Life Sciences, Rochester Institute of Technology, Rochester, NY 14623.
[3] NYU Langone Health, CryoEM Core Facility, Division of Advanced Research Technologies, New York, NY 10016
[4] Advanced Imaging Core, NIDCD, NIH, Bethesda, MD 20892.
[5] NCI, NIH, Frederick, MD 21701.
[6] Electron Microscopy Core Imaging Facility, University of Maryland School of Dentistry, Baltimore, MD 21201.
† Deceased

Materials and Methods

Preparation of capsids for electron microscopy

The day following the infection, the clarified supernatant from the non-permissive *245*(am59) infection, called the "8k supe", was loaded (500 µl) on to a sucrose gradient (10 steps from 20-60%) in PBS (Hoefer) made in a 5 ml Beckman ultra clear tube (cat.# 344057) and centrifuged at 22k for 1 hour at 4°C (Beckman SW28 rotor). Light scattering bands were observed and separately collected by aspiration from the top. In order to remove sucrose, the collected samples were separately dialyzed against 1 liter of PBS overnight in the cold using Slide-a-Lyzers with a 10k MWCO (Pierce cat # 66425). The two fractions were then separately concentrated using Centricon filtration units with 10k MWCO (Amicon ultra15-cat. # 901024). Final sample concentrations were approximately 2 mg/ml. The fractions were then analyzed by SDS-PAGE using a 4-12% Novex tris/glycine gradient gel (Thermo Fisher cat# XPO4120BOX) with MES running buffer (pH, composition), with See-Blue plus2 Protein markers (Thermo Fisher) and stained with Coomassie Blue. Samples were then negatively stained with uranyl acetate (1 %) and imaged using a Philips CM120 Electron Microscope at 120 kV.

Glutaraldehyde solutions (25% - Electron Microscopy Sciences) ranging from 0.3 – 200 mM (10 solutions) were prepared in PBS buffer. For each concentration, 20 µl 8k supe was added to 100 µl glutaraldehyde solution and incubated at room temperature for 15 min. The reaction was stopped by adding 25 µl 1M Tris-HCl (pH 8.0), followed by centrifugation at 3k for 5 min to remove large aggregates. At the highest glutaraldehyde concentrations (>3 mM), we observed large pellets indicating excessive cross-linking and these were discarded. The contents of the other tubes were loaded separately unto 600µl, 20% -50% sucrose 10-step gradients made in PBS and centrifuged in a SW55Ti Beckman rotor at 22k for 45 minutes at 4°C. After centrifugation the 6 gradients were examined under a high intensity lamp. The tubes with a 3.0 mM and 1.5 mM glutaraldehyde

concentration had only a single thick light scattering band located midway in the gradient. The bands were collected by top aspiration, diluted and examined by EM and PAGE.

Electron microscopy

For intact virions we applied 3 µl suspension to a Quantifoil grid with 2 µm holes (Quantifoil, Großlöbichau, Germany) and plunge-froze it into liquid ethane in a KF80 instrument (Reichert-Jung). We imaged the grids in a CM200-FEG microscope (Thermo Fisher, FEI, Hillsboro, OR) operated at 120 keV and a nominal magnification of 38,000x (Table S1). The images were recorded on SO-163 film (Kodak) and scanned on a Nikon Super Coolscan 9000 ED film scanner to give a pixel size of 1.67 Å. For bubblegram imaging, multiple exposures of 0.5 sec 30 seconds apart were taken, each delivering a dose of ~12 e^-/Å^2.

For capsids prepared from the 8k supe, we applied 3 µl suspension (~2 mg/ml) to 40s glow-discharged 400 mesh copper electron microscopy grids with an ultrathin thin carbon film over a lacy carbon support film (Ted Pella, Redding, CA), and plunge-froze it into liquid ethane in a Leica EM GP instrument (Leica Microsystems, Buffalo Grove, IL). We imaged the grids in a 200 kV JEOL 2000FS (JEOL, Peabody, MA) with an in-column energy filter and a K2 Summit direct detection camera (Gatan, Warrendale, PA) for the first data set in counting mode. Subsequent data were acquired with a 300 kV FEI Titan Krios microscope (Thermo Fisher, FEI, Hillsboro, OR) equipped with a post-column Model 967 GIF Quantum-LS and a K2 Summit direct detection camera (Gatan, Warrendale, PA). The micrographs were taken as series of dose-fractionated images and stored without gain correction (Table S1).

Image processing

Data set 1 was processed with EMAN2 [1]. The micrographs were binned twofold and particles were boxed and extracted at a size of 576x576 pixels. An initial reference map was generated using common lines with icosahedral symmetry, followed by 9 iterations of orientation refinement.

All processing for data sets 2-5 was performed using Bsoft [2] and the Peach system to distribute jobs across a cluster [3]. The images were gain-corrected by multiplying with the gain reference (program **bop**) and binned two-fold (program **bint**). The frames were aligned to yield a shift-compensated average for each micrograph (program **bseries**). A power spectrum was calculated for each micrograph as the average over tiles of size 512 x 512 (program **bctf**). The CTF was automatically fitted to the radial power spectrum (program **bctf**). These CTF fits were used to correct the particle images during reconstructions.

A small set of particles were manually picked from the micrographs of the cell lysate (data set 2) imaged on the JEOL microscope and extracted to a size of 380x380 with the program **bpick** (Table S1). The initial orientations were determined using the program **bmaxpow**. In brief the program **bmaxpow** calculates a set of reconstructions from each particle, choosing the orientation that gives greatest power in the reconstruction – effectively a test of coherence for each orientation of the particle. The particles were then incorporated into a full reconstruction using the program **breconstruct**, yielding a resolution of $FSC_{0.3}$ = 70 Å. Using this map as starting template, the global orientations of the particles were determined with the program **borient** through five iterations, and further refined with the program **brefine** for four iterations, producing a final map at $FSC_{0.143}$ =30 Å.

Micrographs from the Krios of the lightly fixed sample (data set 3) were preprocessed as for the JEOL micrographs and particles manually picked (Table S1). The micrographs were split into two sets and each set independently processed. The previous map was used as initial template for the global alignment with the program **borient**, and orientations were refined with the program **brefine** for 8 iterations, with the final alignment performed to a resolution limit of 30 Å. A series of reconstructions were calculated from different numbers of the top-scoring particles. The map composed of the top 2000 particles showed the best resolution of $FSC_{0.143}$ =20 Å (Figure S4) and was filtered to 15 Å with the program **bfilter** for further analysis. The particle view distribution shows some deficiency in 5-fold views (Figure S4B).

The final two sets of micrographs from the Krios (data sets 4 and 5, band 2 and band 3 from the gradient fractionation) were preprocessed as for the JEOL micrographs and particles manually picked (Table S1). Global alignment was performed with the program **borient**, followed by orientation refinement with the program **brefine** for 2 iterations. A series of reconstructions were calculated from different numbers of the top-scoring particles in each case to find the best reconstruction as defined by resolution. The map composed of the top 1000 particles from data set 4 showed the best resolution of $FSC_{0.143}$ =19 Å (Figure S4) and was filtered to 15 Å with the program **bfilter**. The map composed of the top 3000 particles from data set 5 showed the best resolution of $FSC_{0.143}$ =16 Å (Figure S4) and was filtered to 12 Å with the program **bfilter**. The particle view distributions of the two maps show some lack of 5-fold views for the empty capsids (Figure S4D), much less so for the mottled capsids (Figure S4C). The local resolution was calculated with the program **blocres** using a box edge size of 40 pixels (140 Å) (Figure S6) [4].

Internal volume calculation

The volume enclosed by the outer shell was calculated using the program **bsegment**. Briefly, the algorithm floods the inside of the map at a given threshold until it butts up against the outer shell, periodically writing a mask encoding the current content. The mask at the point just before the flooding starts to exit the outer shell boundary is taken as representative of inner volume. The internal volume of the fixed mottled capsid map was estimated at 8.6×10^8 $Å^3$.

Symmetry-adjusted normalized radial profiles

The symmetry-adjusted radial distance was calculated for flat 3-fold faces of the icosahedron, with adjusting the radial distance to correspond to that at the 3-fold axis (program **bradsec**). The profiles were normalized by setting the peak at 580 Å corresponding to the outer capsid shell to one. The peaks for the inner shell were integrated between minima and the total mass calculated relative to the outer shell mass, taken as 136 MDa (including compensating for the change in volume with radius).

Capsid content distribution

The strategy for quantifying the densities in a map was described by Heymann [2]. Briefly, a mask was generated composed of three regions: inside the outer capsid shell, the outer capsid shell and a background region with volumes of 15.9%, 4.6% and 10.6% of the reconstruction volume (5.41×10^9 $Å^3$), respectively. An icosahedral reconstruction was generated for each particle (program bspr) and the densities calculated for each region of the mask. The relative density of the content inside the shell is then calculated as:

$$\rho_{rel} = \frac{d_{inside} - d_{background}}{d_{capsid} - d_{background}}$$

Given an outer capsid shell mass of M_{capsid}, the content mass is given by:

$$M_{inside} = M_{capsid}\rho_{rel}\frac{V_{inside}}{V_{capsid}}$$

For a capsid mass of 136 MDa, the content mass is $461\rho_{rel}$.

Map segmentation 1: The outer shell

The following segmentation was done on the final map of each of the three reconstructions (mottled, mottled-fixed and empty) filtered to 15 Å. The first task was to accurately locate the capsomers in the outer shell to allow their extraction, averaging and building a representation of the outer shell. Using the modeling tools in the program **bshow**, 5 components were placed at the centers of the 5 hexameric capsomers of the outer capsid shell. These were extracted and averaged into a single map using the program **bxb**. The average was six-fold symmetrized using the program **bsym**. To isolate only the capsomer in the symmetrized map, it was further segmented with the program **bflood** (a watershed algorithm), the segment corresponding to the capsomer extracted and converted to a mask that was then applied to the original symmetrized average. This map was used as reference to refine the positions of the capsomers with the program **bxb**, followed by extraction, averaging, symmetrization, segmentation and masking as before to generate a new reference capsomer map. After three iterations, a sixth component was placed at the pentameric capsomer at the five-fold vertex and labelled separately from the hexameric capsomers. The locations of the capsomers were further refined for two more iterations with two reference maps. The final model was icosahedrally symmetrized to generate all the capsomeric components. The two masked averages from the last iteration were then used to build a full icosahedral outer capsid shell.

Map segmentation 2: The inner shell

The outer capsid shell map constructed as described in the previous sections was subtracted from the full map to reveal the inner densities. Examining the inner map, we located 12 similar-looking densities with a helical twist in one asymmetric unit. Markers were placed at these locations in the program Chimera [5], exported to a .cmm file and converted to the .star format used in Bsoft with the program **bmodel**. One of these densities was selected as reference, segmented using the program **bflood** and the desired density isolated as for the capsomers. This was used as reference to determine and refine the locations and orientations of all the densities for 4 iterations using the program **bxb** as for the capsomers in the previous section. The final model was icosahedrally symmetrized and a full icosahedral map built. This map was subtracted from the inner densities map, producing a map that shows the remaining densities.

Image analysis of the 8k supe sheets

Areas showing lattices were extracted from electron micrographs of data set 2 taken on the JEOL microscope. One such lattice (Figure 6A) was Fourier transformed to obtain its diffraction pattern in the program bshow (Figure 6B), the spots masked, and backtransformed to produce a filtered lattice (Figure 6C). One unit cell was extracted from this lattice as reference for single particle analysis. The reference was used to identify 52179 unit cells from 19 sheets, each extracted into a "particle" image. These were aligned to the reference and averaged through five iterations, yielding

4 class averages. The class averages appear to represent slightly different views of the unit cell, suggesting that the sheets are not perfectly flat and perpendicular to the electron beam. One of these averages (from 7385 unit cells) show a very regular square lattice (Figure 6D) consistent with the 66.8 Å spacing in Figure 6B. Analysis of the SSNR of this class average indicated a resolution of ~24 Å.

Table S1: Image processing report for the DNA-filled mature capsid (Mat), the mottled capsid (Mott) in the clarified cell lysate, glutaraldehyde-fixed and unfixed, and the empty outer capsid.

Parameter	WT head (Data set 1)	Mott (Data set 2)	Mott (fixed) (Data set 3)	Empty (unfixed) (Data set 4)	Mott (unfixed) (Data set 5)
EMDB code			EMD-22332	EMD-22331	EMD-22333
Micrographs:					
Microscope	CM200	JEOL 2200FS	Krios G3	Krios G3	Krios G3
Energy filter	No	In-column	GIF	GIF	GIF
Camera	Film	K2, counting	K2, superres	K2, counting	K2, counting
Magnification	38000	15000	81000	81000	81000
Pixel size (Å)	1.67	2.39	1.76†	1.76	1.76
Number	12	271	3479	1178	2244
Frames per micrograph	1	54	100	60	60
Frame rate (/s)	2	2	10	4	4
Dose per frame (e^-/pixel)	nd	3.7	0.9†	2.3	2.3
Accumulated dose (e^-/Å^2)	nd	35	29	138	138
Frame alignment	na	bseries	bseries	bseries	bseries
CTF:					
Defocus range (µm)	2.3 – 2.7	1.73 – 2.75	1.22 – 3.5	0.49 – 2.69	0.57 – 3.1
Correction	Phase flip	Phase flip	Phase flip	Phase flip	Phase flip
Particles:					
Size (pixels)	576x576	380x380	500x500	500x500	500x500
Pixel size (Å)	3.34	4.78	3.51	3.51	3.51
Picked (manually)	119	423	8633	1814	4440
Used in final 3D reconstruction	119 (100%)	365 (86%)	2000 (23%)	1000 (55%)	3000 (68%)
Alignment:					
Initial reference map	EMAN2	bmaxpow	*	**	**
Low-pass filter limit (Å)	25	30	25	40	40
Algorithm	Common line	Cross-corr.	Cross-corr.	Cross-corr.	Cross-corr.
Number of iterations	9	25	9	3	3
Reconstruction:					
Size	576x576x576	380x380x380	500x500x500	500x500x500	500x500x500
Pixel size (Å)	3.34	4.78	3.51	3.51	3.51
Symmetry	I	I	I	I	I
Resolution limit (Å)	7	10	7	7	7
Resolution est. (Å, $FSC_{0.143}$)	~30	30	20	19	16
Masked	no	yes	yes	yes	yes
Sharpened	no	no	no	no	no
Low-pass filter limit	25 Å	25 Å	15 Å	15 Å	12 Å

† The micrographs were taken in super-resolution mode, subsequently binned for further processing (final size: 3838 x 3710).

* Initial map derived from the final map from set 1 (column 2)

** Initial map derived from the final map from set 2 (column 3)

nd: not done; na: not applicable

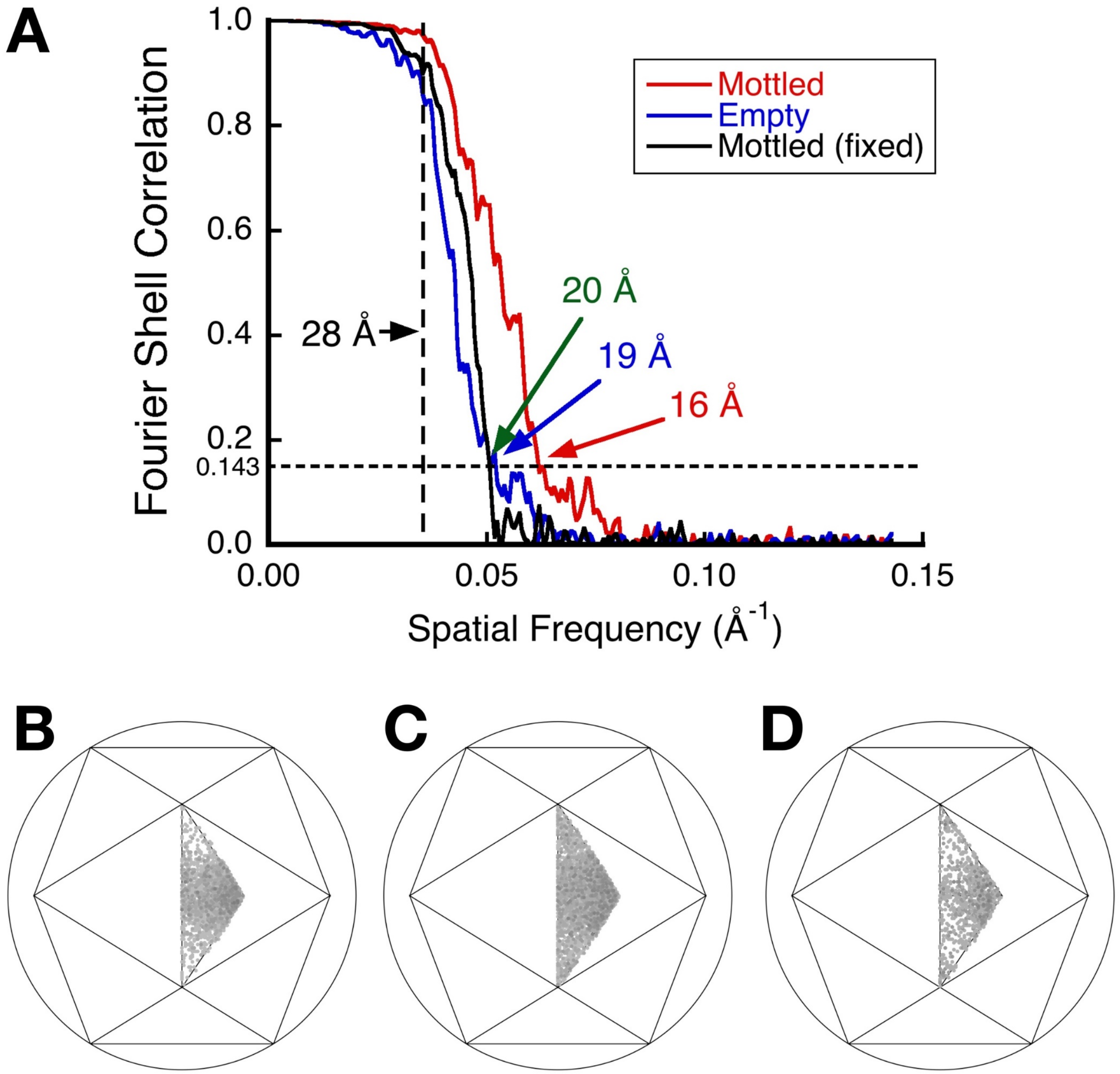

Figure S1: Reconstruction details. (A) Fourier shell correlation for the mottled (red), mottled fixed with glutaraldehyde (green) and empty (blue) capsid reconstructions. The vertical dashed line at 28 Å resolution indicates the final resolution cutoff used to align the particles. Particle view distributions for (B) the glutaraldehyde-fixed mottled capsids (2000 particles), (C) the non-fixed mottled capsids (3000 particles), and (D) the empty capsids (1000 particles).

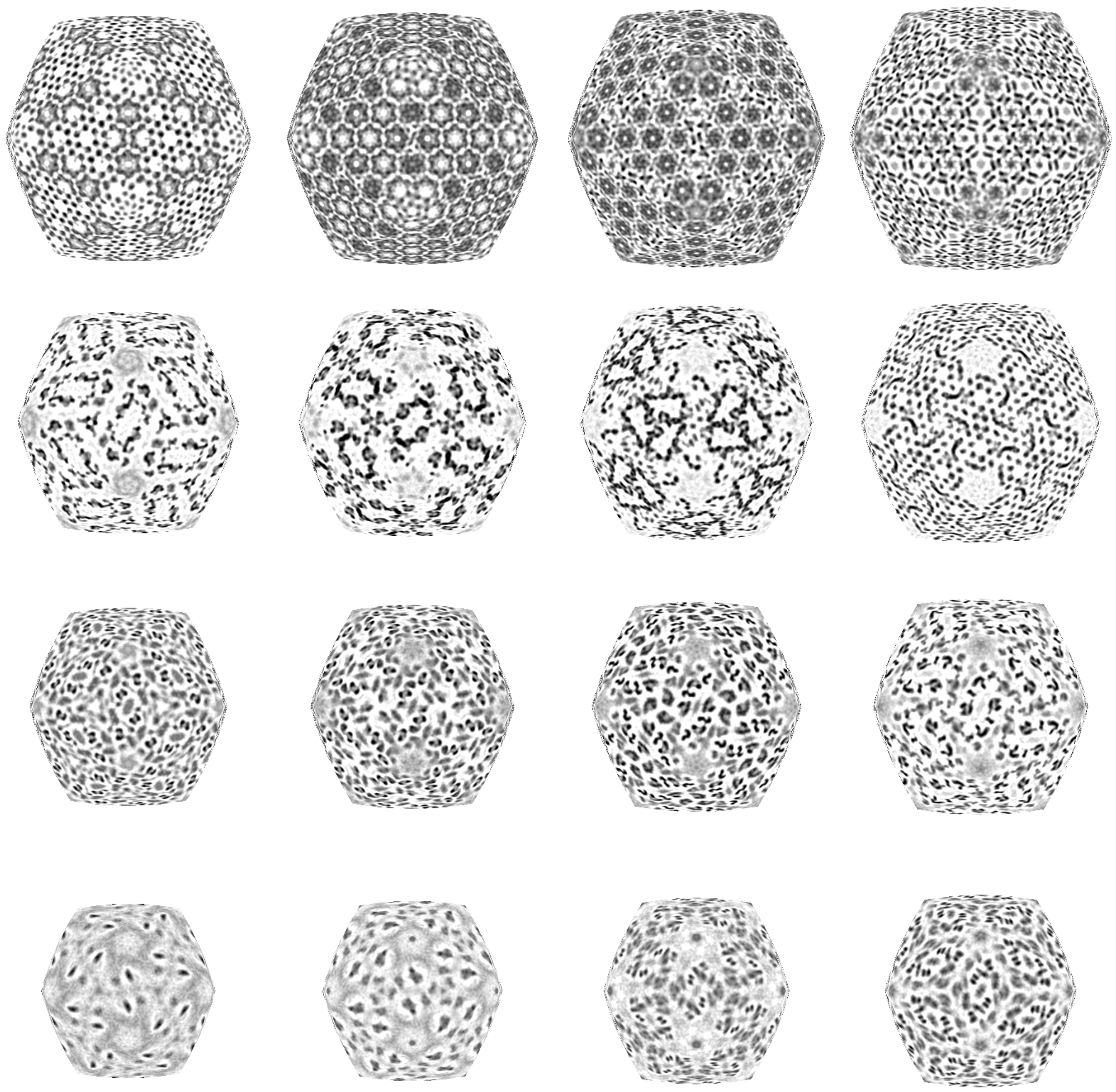

Figure S2: Icosahedral-adapted spherical sections of the fixed mottled capsid reconstruction, starting at a radius of ~410 Å (lower left) and ending at ~620 Å (upper right) with ~14 Å steps.

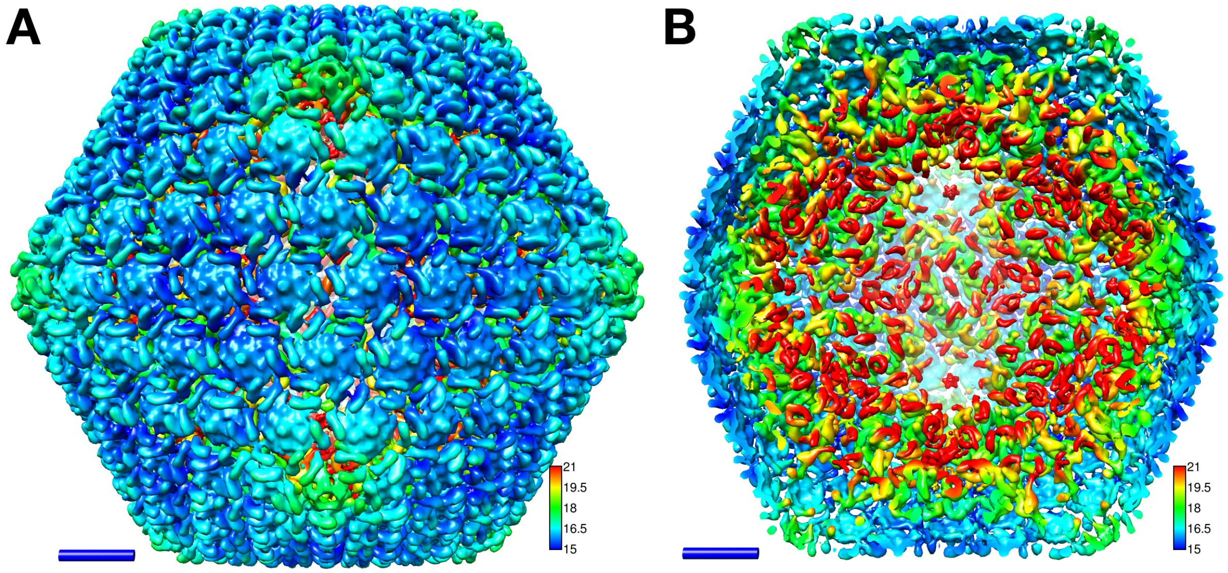

Figure S3: Isosurfaces of the unfixed mottled capsid reconstruction colored by local resolution estimates. (A) Outside view, showing the T=27 icosahedral outer shell. (B) Cut through to show the T=1 icosahedral inner shell, decreasing in local resolution towards the middle. Many twisted hook-like densities are evident, likely many copies of the ejection proteins. Scale bars: 200 Å.

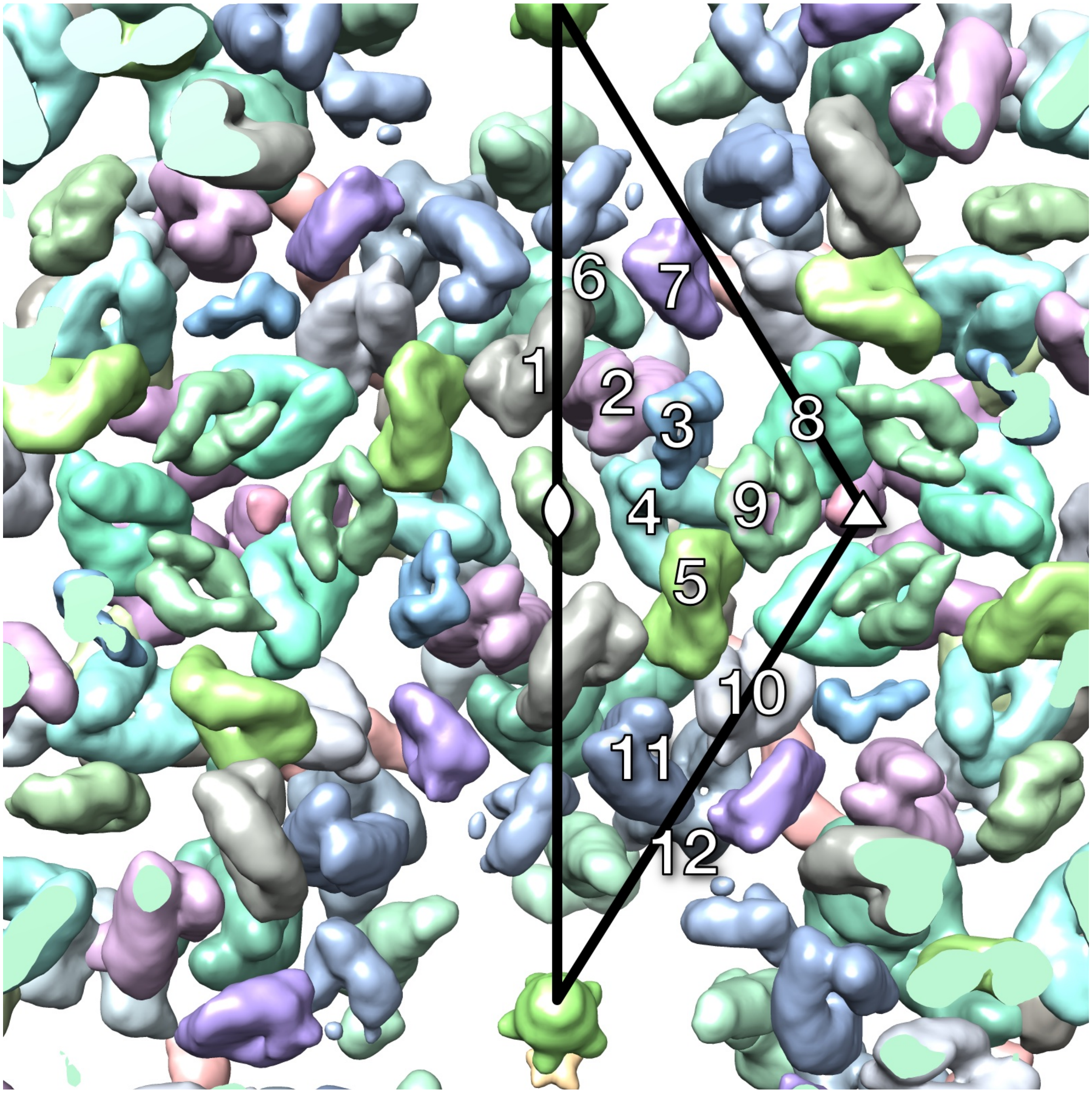

Figure S4: Segmentation of the inner shell of the fixed mottled capsid reconstruction, with symmetry-related densities colored the same. The large black triangle follows the borders of an asymmetric unit. The numbers indicate twisted hook-like densities varying in apparent size – more a reflection of thresholding densities with varying flexibility rather than apparent mass. (Thanks to Greg Pintilie for modifications to the Segger plugin for Chimera to color symmetry-related densities [6]).

References

1. Tang, G.; Peng, L.; Baldwin, P.R.; Mann, D.S.; Jiang, W.; Rees, I.; Ludtke, S.J. EMAN2: an extensible image processing suite for electron microscopy. *J Struct Biol* **2007**, *157*, 38-46.
2. Heymann, J.B. Guidelines for using Bsoft for high resolution reconstruction and validation of biomolecular structures from electron micrographs. *Protein Sci* **2018**, *27*, 159-171, doi:10.1002/pro.3293.
3. Leong, P.A.; Heymann, J.B.; Jensen, G.J. Peach: a simple Perl-based system for distributed computation and its application to cryo-EM data processing. *Structure* **2005**, *13*, 505-511.
4. Cardone, G.; Heymann, J.B.; Steven, A.C. One number does not fit all: mapping local variations in resolution in cryo-EM reconstructions. *J Struct Biol* **2013**, *184*, 226-236, doi:10.1016/j.jsb.2013.08.002.
5. Pettersen, E.F.; Goddard, T.D.; Huang, C.C.; Couch, G.S.; Greenblatt, D.M.; Meng, E.C.; Ferrin, T.E. UCSF Chimera--a visualization system for exploratory research and analysis. *J Comput Chem* **2004**, *25*, 1605-1612.
6. Pintilie, G.D.; Zhang, J.; Goddard, T.D.; Chiu, W.; Gossard, D.C. Quantitative analysis of cryo-EM density map segmentation by watershed and scale-space filtering, and fitting of structures by alignment to regions. *J Struct Biol* **2010**, *170*, 427-438, doi:10.1016/j.jsb.2010.03.007.
